# Preemptive flurbiprofen axetil for sleep-pain-inflammation modulation after laparoscopic gynecological surgery: a prospective, parallel-group randomized controlled trial

**DOI:** 10.3389/fphar.2025.1659179

**Published:** 2025-10-07

**Authors:** Xin Huang, Wenxin Wei, Zhihao Leng, Bijia Song, Ming Fu, Jiangshan He, Junchao Zhu

**Affiliations:** ^1^ Department of Anesthesiology, Union Hospital, Tongji Medical College, Huazhong University of Science and Technology, Wuhan, China; ^2^ Department of Anesthesiology, Shengjing Hospital of China Medical University, Shenyang, China; ^3^ Department of Anesthesiology, The Fourth People’s Hospital of Shenyang, Shenyang, China; ^4^ Department of Anesthesiology, The Central Hospital of Wuhan, Tongji Medical College, Huazhong University of Science and Technology, Wuhan, China; ^5^ Department of Anesthesiology, Beijing Friendship Hospital of Capital Medical University, Beijing, China

**Keywords:** flurbiprofen axetil, preemptive analgesia, postoperative pain, sleep quality, systemic inflammation, laparoscopic gynecological surgery, nonsteroidal anti-inflammatory drugs (NSAIDs)

## Abstract

**Background:**

Postoperative pain, inflammation, and sleep disturbances commonly arise after laparoscopic gynecological surgery and are increasingly recognized as interconnected factors that impede recovery and diminish quality of life. Flurbiprofen axetil, classified as a NSAID, is widely used during the perioperative period for pain management. Its potential to modulate inflammatory pathways and nociceptive transmission, thereby enhancing postoperative sleep quality, remains underexplored. Additionally, the optimal timing for NSAID administration—preoperative versus postoperative—remains debated, with limited evidence addressing its impact on sleep outcomes.

**Objectives:**

This study investigates the impact of preoperative compared to postoperative use of flurbiprofen axetil on pain, systemic inflammatory responses, and particularly the sleep quality in patients undergoing laparoscopic gynecologic operations.

**Methods:**

In this prospective, randomized controlled trial, 98 patients undergoing laparoscopic gynecological surgery were assigned to receive 50 mg of flurbiprofen axetil either 15 min prior to surgery (PreFA group) or at the end of surgery (PostFA group). The primary outcomes assessed included postoperative pain intensity (visual analog scale, VAS), sleep quality (Athens Insomnia Scale, AIS), and systemic inflammatory markers (SII, NLR, and MLR). Outcome data were collected by blinded assessors at predefined time points: preoperatively, 24 h, and 72 h postoperatively.

**Results:**

The baseline characteristics were similar between groups. The PreFA group demonstrated significantly lower VAS scores for both resting and exertional pain at 24 and 72 h after surgery (P < 0.05). AIS scores were also substantially lower in this group on postoperative days 1 and 3 (P < 0.001 and P = 0.002), reflecting improved sleep quality. Inflammatory markers (SII, NLR, MLR) were elevated postoperatively in both groups but remained significantly lower in the PreFA group (all P < 0.05). Additionally, the incidence of postoperative nausea and vomiting was reduced in the PreFA group.

**Conclusion:**

Preoperative administration of flurbiprofen axetil not only improved postoperative analgesia and reduced inflammatory responses but also significantly enhanced sleep quality, an essential yet frequently underestimated component of recovery. These findings underscore the broader physiological benefits of preemptive NSAID use and emphasize the importance of timing in analgesic strategies. Incorporating flurbiprofen axetil into preemptive multimodal analgesia protocols could provide a straightforward yet effective approach to optimizing recovery following laparoscopic gynecological surgery.

**Clinical Trial Registration:**

clinicaltrials.gov, identifier NCT04611763.

## 1 Introduction

Gynecological disorders are increasingly prevalent globally, driven by both environmental and lifestyle factors (Piechocki et al., 2022; Berghuis et al., 2022; Louie et al., 2018; Buia et al., 2015; Vennix et al., 2014). Laparoscopic surgery has become the standard approach for treating many of these conditions, due to its minimal invasiveness, faster recovery, and shorter hospital stays (Louie et al., 2018). Nevertheless, patients continue to experience considerable postoperative pain and sleep disturbances, which can impede recovery, prolong hospitalization, and deteriorate life quality (Qiu et al., 2022; Rampes et al., 2019).

A major contributor to these symptoms is the use of carbon dioxide (CO_2_) pneumoperitoneum in laparoscopic procedures, which irritates the peritoneum and diaphragm, activates peripheral nociceptors, and triggers inflammatory mediator release (Ji et al., 2018). This process often results in diffuse, non-incisional pain, such as referred shoulder or upper abdominal pain, that may even surpass incisional discomfort in severity (Hsien et al., 2017; Mouton et al., 1999; Shin et al., 2010; Phelps et al., 2008; Slim et al., 1999; Sao et al., 2019; Lee et al., 2018). Importantly, pain and sleep disturbances are interdependent in a bidirectional manner: pain disrupts sleep continuity and architecture, while impaired sleep lowers pain thresholds and amplifies nociceptive sensitivity, forming a vicious cycle that delays recovery (Chouchou et al., 2014; Miller et al., 2015).

Surgical trauma further induces a systemic inflammatory response, which contributes to both pain hypersensitivity and sleep dysregulation. Biomarkers such as the systemic immune-inflammation index (SII), neutrophil-to-lymphocyte ratio (NLR), monocyte-to-lymphocyte ratio (MLR), and C-reactive protein (CRP) have been validated as sensitive indicators of this inflammatory state (Moldovan et al., 2023; Xing et al., 2024; Xing et al., 2023; Marver and McGlinchey, 2020; Polyné et al., 2021) Furthermore, increased levels of cytokines like IL-6 and CRP have been independently linked to postoperative sleep disturbance, suggesting that inflammation may be a critical mechanistic pathway linking pain and impaired sleep (Irwin and Opp, 2017).

Given this shared pathophysiology, an analgesic strategy that concurrently attenuates both pain and systemic inflammation may hold promise for improving postoperative sleep quality. Flurbiprofen axetil, a non-steroidal anti-inflammatory drug (NSAID) with both central and peripheral mechanisms of action, is well-suited for this purpose. With high affinity for inflamed tissue, it inhibits prostaglandin synthesis and neural hyperexcitability, providing prolonged, targeted analgesia (Marver and McGlinchey, 2020).

Evidence suggests that flurbiprofen axetil plays an effective role in managing postoperative pain, minimizing opioid use, and relieving common opioid-induced complications such as nausea, vomiting, and respiratory depression, all of which may contribute to sleep problems (Nowakowski and Meers, 2019; Chung et al., 2014). The concept of preemptive analgesia—administering analgesics prior to surgical insult—has been widely advocated as a strategy to prevent central sensitization and enhance postoperative recovery (Polyné et al., 2021). Although preoperative NSAID administration is supported by theoretical rationale, recent studies have reported limited efficacy in reducing pain intensity or opioid consumption, often without direct comparisons to postoperative use (In et al., 2023). Notably, the impact of timing on postoperative sleep quality—particularly in relation to flurbiprofen axetil—remains underexplored, especially in the setting of laparoscopic gynecological surgery.

This prospective randomized controlled trial aimed to evaluate the clinical outcomes of preoperative versus postoperative administration of flurbiprofen axetil on postoperative pain levels, systemic inflammation, and sleep quality. Given the close interplay between pain, inflammation, and sleep, we hypothesized that preemptive use of flurbiprofen axetil may confer superior multidimensional recovery benefits, offering new insights for optimizing perioperative NSAID protocols.

## 2 Methods

### 2.1 Patient recruitment

Ethical approval for this study was granted by the Human Research Ethics Committee of Shengjing Hospital, China Medical University (IRB No. 2022PS1115K). This trial was registered at ClinicalTrials.gov (Unique Identifier: NCT04611763, clinicalTrials.gov, https://clinicaltrials.gov/study/NCT04611763) before participant enrollment. Written informed consent was obtained from all participants before inclusion. The study was conducted in accordance with the ethical principles outlined in the Declaration of Helsinki.

#### 2.1.1 Participants

This study enrolled patients undergoing elective laparoscopic gynecological surgery under general anesthesia at Shengjing Hospital, China Medical University. Eligible participants were required to meet the following inclusion criteria: aged 18–75 years; classified as American Society of Anesthesiologists (ASA) physical status I or II; no prior neoadjuvant or adjuvant chemotherapy (Excluded due to potential influence on baseline inflammation. Previous studies have shown that both neoadjuvant and adjuvant chemotherapy can alter inflammatory markers such as the neutrophil-to-lymphocyte ratio (NLR), systemic immune-inflammation index (SII), and platelet-to-lymphocyte ratio (PLR). For example, Mleko et al. (2023) reported dynamic decreases in SII, NLR, and PLR during adjuvant paclitaxel–carboplatin chemotherapy in epithelial ovarian cancer. Similarly, Sanna et al. (2021) demonstrated that a decrease in NLR during neoadjuvant chemotherapy was significantly associated with clinical response, suggesting that chemotherapy-related immune changes may influence systemic inflammation markers); no use of medications that could affect hematological parameters preoperatively; no history of acute or chronic inflammatory diseases; no use of anti-inflammatory or immunosuppressive drugs within the last 3 months; and normal liver function. Exclusion criteria included a history of central nervous system or psychiatric disorders; preexisting sleep disorders; current or past use of sedatives, analgesics, or antidepressants; diagnosis of sleep apnea or moderate to severe obstructive sleep apnea-hypopnea syndrome (OSAHS); history of chronic gastritis or gastric ulcer; known allergy to flurbiprofen axetil; impaired communication abilities; or unwillingness to participate in the trial.

The sample size was initially calculated to be 74 participants. A pre-experimental pilot study was conducted involving 40 patients, with 20 allocated to the preoperative flurbiprofen axetil group (PreFA group) and 20 to the postoperative administration group (PostFA group). The primary outcome was postoperative pain intensity at 72 h, measured using the visual analog scale (VAS). The mean VAS scores were 1.70 ± 0.57 in the PreFA group and 2.35 ± 0.75 in the PostFA group.

Based on these data, the effect size (Cohen’s d) was estimated to be approximately 0.98.

The sample size estimation was performed using PASS software version 15.0 (NCSS, LLC, Kaysville, UT, United States). Assuming a two-sided alpha level of 0.05 and a power (1 − β) of 95%, a minimum of 29 participants per group was required to detect a significant difference in VAS scores. To account for an anticipated 20% dropout rate,the sample size was increased to 37 participants per group, resulting in a total target enrollment of 74 participants.

This effect size was directly derived from pilot data and reflects a clinically meaningful difference in postoperative pain scores between the two groups.

**FIGURE 1:**
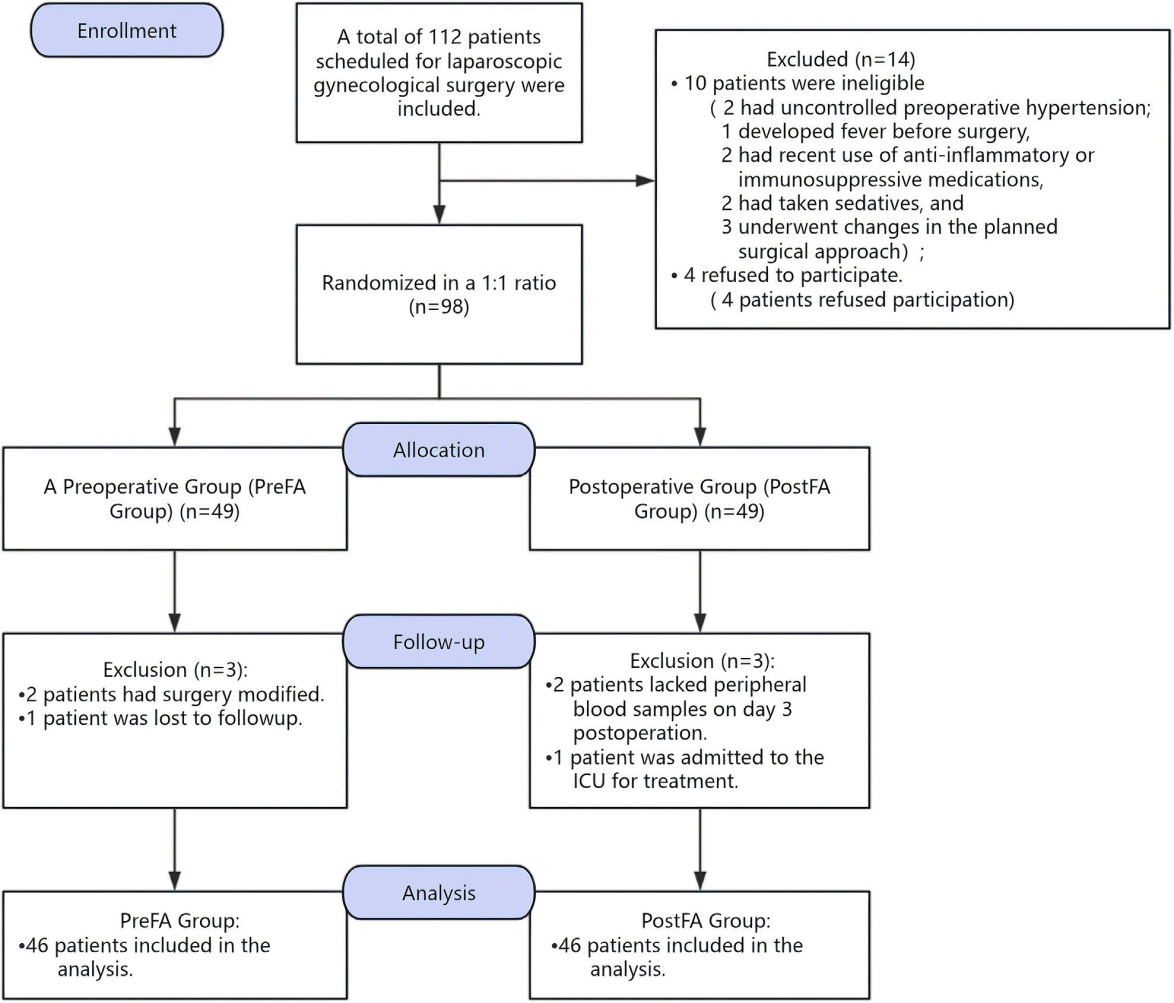
Patient screening and inclusion flowchart.

However, a total of 112 participants were enrolled during the pre-anesthesia evaluation conducted 1 day prior to randomization. Although the planned sample size was 74 patients, including a 20% allowance for potential post-randomization dropouts, we intentionally over-recruited based on our center’s prior clinical experience. Specifically, preoperative changes—such as elevated blood pressure, fever, or other conditions requiring surgical cancellation or conversion to open abdominal surgery—frequently resulted in patient ineligibility prior to randomization. To mitigate these anticipated exclusions, approximately 20%–30% more patients were recruited. Ultimately, 92 patients completed the study and were included in the per-protocol (PP) analysis. The PP population comprised 46 patients in the PreFA group and 46 in the PostFA group, all of whom completed the required interventions and outcome assessments without major protocol deviations. This PP analysis was conducted to evaluate the treatment effect under ideal conditions, with strict protocol adherence. Given the final sample size exceeded the minimum requirement, and all PP participants completed the study as intended, we proceeded with a per-protocol analysis. A detailed participant flow, including pre-randomization exclusions and any post-randomization dropouts, is presented in Figure 1, per CONSORT guidelines. All statistical analyses were conducted with a two-sided P-value < 0.05 considered statistically significant.

### 2.2 Statistical analyses

All statistical analyses were performed using SPSS version 26.0 (IBM Corp., Armonk, NY, USA) and GraphPad Prism version 9.5.

The normality of continuous variables was assessed using the Shapiro–Wilk test, supplemented by the D’Agostino–Pearson omnibus test, Anderson–Darling test, and Kolmogorov–Smirnov test. Variables with P > 0.05 were considered normally distributed. Normally distributed data are presented as mean ± standard deviation (SD), while non-normally distributed data are expressed as median with interquartile range (IQR).

Between-group comparisons were performed using independent-samples t-tests for normally distributed variables with equal variances. Welch’s t-test was applied when variances were unequal, as determined by Levene’s test. For non-normally distributed variables, the Mann–Whitney U test was used.

Categorical variables were summarized as number (n) and percentages (%).

Comparisons between groups were conducted using the chi-squared test or Fisher’s exact test, as appropriate.

A two-tailed P value < 0.05 was considered statistically significant.

### 2.3 Randomization into treatment groups

A total of 98 patients were randomized in a 1:1 ratio to receive flurbiprofen axetil either preoperatively (PreFA group, n = 49) or postoperatively (PostFA group, n = 49) using a computer-generated randomization sequence. Randomization was performed by an independent researcher not involved in patient care or outcome evaluation. Group assignments were concealed in sequentially numbered, opaque envelopes to ensure allocation concealment.

Blinding was maintained for participants, anesthesiologists, surgeons, and outcome assessors, consistent with a quadruple-blind design. Since both groups received the same drug at different time points, the timing of administration was concealed by delivering the medication in the operating room behind surgical drapes. To ensure effective blinding, the drug was administered by a designated anesthesiologist who was not involved in the intraoperative anesthetic management, postoperative care, data collection, or outcome assessment. This procedure ensured that neither the patients nor the intraoperative clinical team were aware of whether flurbiprofen axetil was administered before or after surgery. Sleep quality evaluations were conducted by independent anesthesiologists who were blinded to group allocation. All personnel involved in outcome evaluation and data analysis remained blinded to group assignments until the database was locked for final statistical analysis.

Postoperative sleep quality was assessed using the Athens Insomnia Scale (AIS), a validated, ICD-10-based self-report instrument comprising 8 items with a total score ranging from 0 to 24. A score ≥6 indicates probable insomnia (Okajima et al., 2013; Soldatos et al., 2000; Soldatos et al., 2003). AIS assessments were conducted on the night before surgery (Sleep Preop 1) and on postoperative days 1 and 3 (Sleep POD1 and POD3).

Systemic inflammatory markers, including the Systemic Immune-Inflammation Index (SII), Neutrophil-to-Lymphocyte Ratio (NLR), and Monocyte-to-Lymphocyte Ratio (MLR), were measured in peripheral blood samples collected preoperatively and on postoperative days 1 and 3. Calculations for these markers are detailed below:

SII=platelet count×neutrophil count / lymphocyte count

NLR=neutrophil count / lymphocyte count

MLR=monocyte count / lymphocyte count

Hemodynamic parameters—including mean arterial pressure (MAP) and heart rate (HR), were recorded at the following six timepoints: T0 (5 min after operating room entry), T1 (immediately after intubation), T2 (5 min post-intubation), T3 (end of surgery), T4 (extubation), and T5 (5 min post-extubation).

General anesthesia was induced with intravenous propofol (2 mg/kg), nalbuphine hydrochloride (0.2 mg/kg), and rocuronium bromide (0.6 mg/kg). Tracheal intubation was performed, followed by mechanical ventilation, with tidal volume and respiratory rate adjusted to maintain end-tidal carbon dioxide (EtCO_2_) between 35 and 45 mmHg.

Anesthesia was maintained via a combination of intravenous and inhalational agents. Propofol (4–8 mg/kg/h) and remifentanil (0.1–0.2 µg/kg/min) were administered intravenously. Sevoflurane (0.5%–2%) was inhaled to maintain a minimum alveolar concentration (MAC) ≥ 0.7, along with 100% oxygen at a fresh gas flow of 2 L/min. Rocuronium bromide (0.2 mg/kg) was administered intermittently to maintain muscle relaxation.

To prevent postoperative nausea and vomiting (PONV), ramosetron hydrochloride (0.3 mg) was administered intravenously 30 min before the end of surgery. All anesthetic agents were discontinued after surgery, and patients were extubated and transferred to the post-anesthesia care unit (PACU) for monitoring.

Postoperative analgesia was managed using a patient-controlled analgesia (PCA) pump containing ramosetron hydrochloride (0.6 mg) and nalbuphine hydrochloride (1 mg/kg), diluted to 100 mL with normal saline. Once patients regained full consciousness (indicated by following commands such as eye opening, finger squeeze, and deep breathing), the PCA pump was activated to deliver 2 mL/h continuously, with a bolus dose of 0.5 mL on demand and a 15-min lockout interval. Total cumulative PCA dosage was recorded at 24 h postoperatively.

Postoperative pain was assessed using a 10-cm visual analogue scale (VAS). Patients were asked to mark their perceived pain intensity on a horizontal line with endpoints labeled “no pain” (0 cm) on the left and “worst imaginable pain” (10 cm) on the right (El Sherif et al., 2016). The distance in centimeters from the left end to the mark was measured and recorded as the VAS score. Assessments were conducted during rest and during exertion, before surgery and again at 24 and 72 h after the procedure.

## 3 Results

Initially, 112 patients were screened for eligibility. Fourteen were excluded due to not meeting the inclusion criteria, and six refused participation. As a result, 92 patients were finally enrolled in the PP group (Figure 1).

### 3.1 Baseline characteristics of the PreFA and PostFA groups

The demographic and perioperative characteristics of patients in the PreFA and PostFA groups were well balanced, with no statistically significant differences observed in age, BMI, duration of surgery, duration of anesthesia, intraoperative blood loss, intraoperative fluid volume, or surgical type (all P > 0.05; Table 1).

**TABLE 1:** Comparison of basic characteristics between the PreFA and PostFA groups.

Characteristic	PreFA Group (n = 46)	PostFA Group (n = 46)	Test value (t-value/χ^2^ value)	P value
Age (years)	42.80 ± 8.51	43.33 ± 9.25	−0.28	0.78
BMI(kg/m^2^)	23.54 ± 2.63	23.98 ± 2.92	−0.76	0.45
Surgical duration (min)	80.91 ± 16.64	84.46 ± 20.05	−0.92	0.36
Anesthesia duration (min)	95.00 (85.00,110.25)	105.00 (89.25,115.00)	−1.21	0.23
Blood loss (mL)	100.00 (50.00,120.00)	100.00 (50.00,150.00)	−0.29	0.77
Intraoperative fluid volume (mL)	700.00 (600.00,900.00)	800.00 (675.00,1100.00)	−1.80	0.07
Surgical type (n, %)	46 (100.00)	46 (100.00)	0.77	0.88
Laparoscopic uterine lesion resection	13 (28.26)	11 (23.91)	0.06	0.81
Laparoscopic total hysterectomy	23 (50.00)	21 (45.65)	0.04	0.84
Laparoscopic total hysterectomy with unilateral adnexectomy	4 (8.70)	6 (13.04)	0.11	0.74
Laparoscopic total hysterectomy with bilateral adnexectomy	6 (13.04)	8 (17.39)	0.08	0.77

Values are presented as mean ± standard deviation or median (interquartile range) for continuous variables, and as number (percentage) for categorical variables.

Test values represent t-values for normally distributed continuous variables, Chi-square tests with Yates’ continuity correction were used for all 2 × 2 categorical variables. P < 0.05 was considered statistically significant.

### 3.2 Intraoperative vital signs between the two groups

**FIGURE 2:**
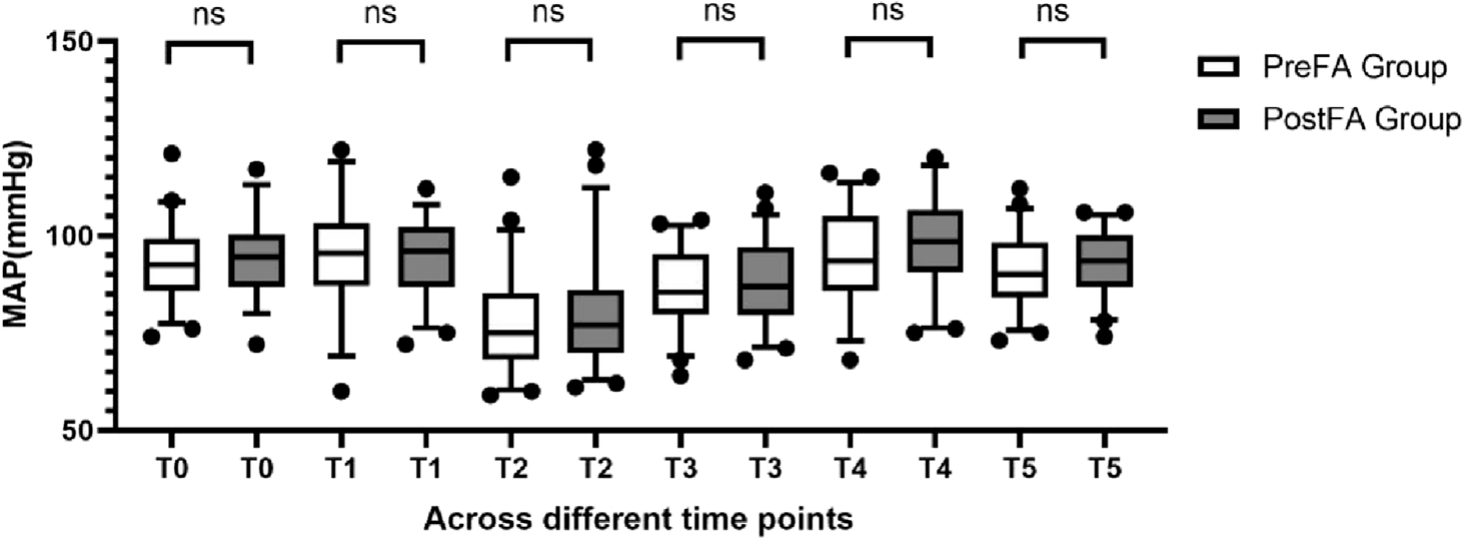
Comparison of MAP at different time points between PreFA and PostFA groups.

**FIGURE 3:**
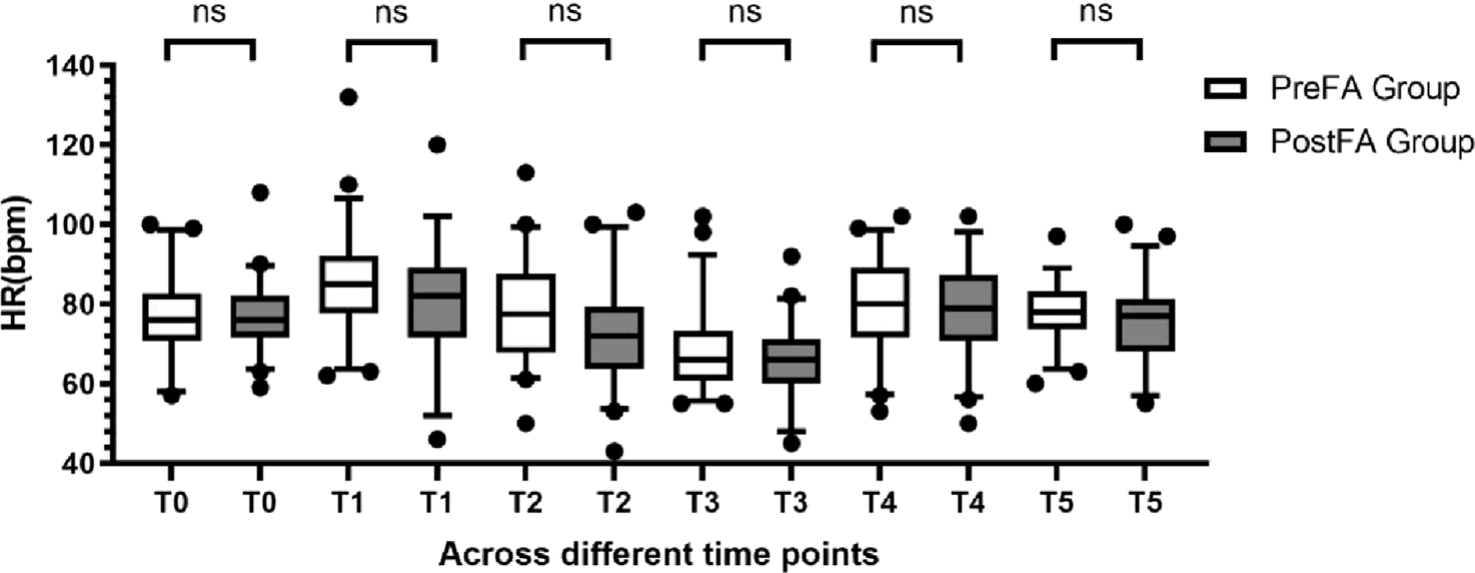
Comparison of HR at different time points between PreFA and PostFA groups.

MAP and HR remained comparable between the two groups at T0 (preoperative) and at each intraoperative time point (T1 to T5; P > 0.05 for all) (Figures 2, 3).

### 3.3 Comparison of postoperative medication and pain outcomes between the PreFA and PostFA groups

**FIGURE 4:**
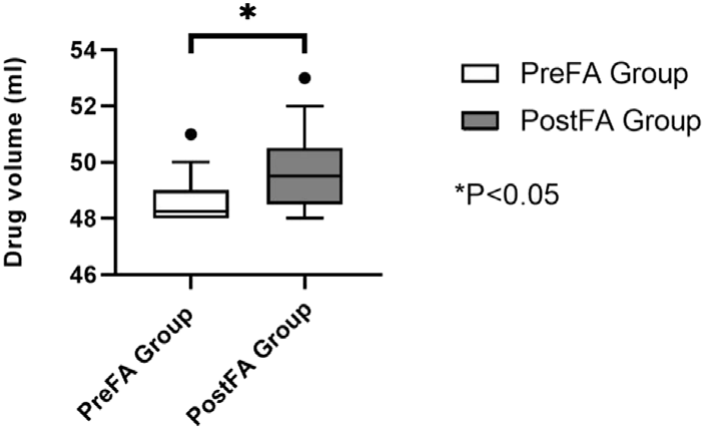
Total dose of analgesic pump medication (mL) used in the first 24 h postoperatively.

Postoperative analgesic consumption within the first 24 h was significantly reduced in the PreFA group compared to the PostFA group (P < 0.05) (Figure 4).

At 24 h after surgery, both resting and coughing pain scores were significantly higher in the PostFA group compared to the PreFA group (P = 0.003 and P < 0.001, respectively). By 72 h, resting pain scores showed no significant difference between the two groups; however, coughing pain remained significantly elevated in the PostFA group (P = 0.001 and P < 0.001, respectively) (Table 2). Additionally, the total volume of analgesic pump medication (mL) used within 24 h postoperatively was markedly greater in the PostFA group than in the PreFA group (P < 0.001).

**TABLE 2:** Comparison of pain scores and analgesic pump medication usage between PreFA and PostFA group.

Pain intensity and total analgesic dose	PreFA Group (n = 46)	PostFA Group (n = 46)	t 值	*P*值
Preoperative Resting pain (VAS Score)	0.00 (0.00,0.25)	0.00 (0.00,0.00)	−0.25	0.81
Preoperative exertional pain (VAS Score)	0.00 (0.00,1.00)	0.00 (0.00,1.00)	−0.61	0.54
Resting pain in the first 24 h postoperatively (VAS Score)	3.00 (2.00,3.25)*	4.00 (3.00,5.00)	−2.99	0.00
Exertional pain in the first 24 h postoperatively (VAS Score)	4.00 (3.00,5.00)*	5.00 (4.00,6.00)	−3.29	0.00
Resting pain in the first 72 h postoperatively (VAS Score)	2.00 (1.00,2.00)*#	2.00 (2.00,3.00)	−3.91	0.00
Exertional pain in the first 72 h postoperatively (VAS Score)	2.00 (2.00,3.00)*#	3.00 (2.75,4.00)	−3.22	0.00
Total dose of analgesic pump medication used in the first 24 h postoperatively (ml)	48.25 (48.00,49.00)	49.50 (48.50,50.50)	−4.15	0.00

*, P < 0.05 when compared to baseline (preoperative); #, P < 0.05 when compared to 24-h postoperative values.

### 3.4 Comparison of SII, NLR, and MLR before and after surgery between the two groups

**FIGURE 5:**
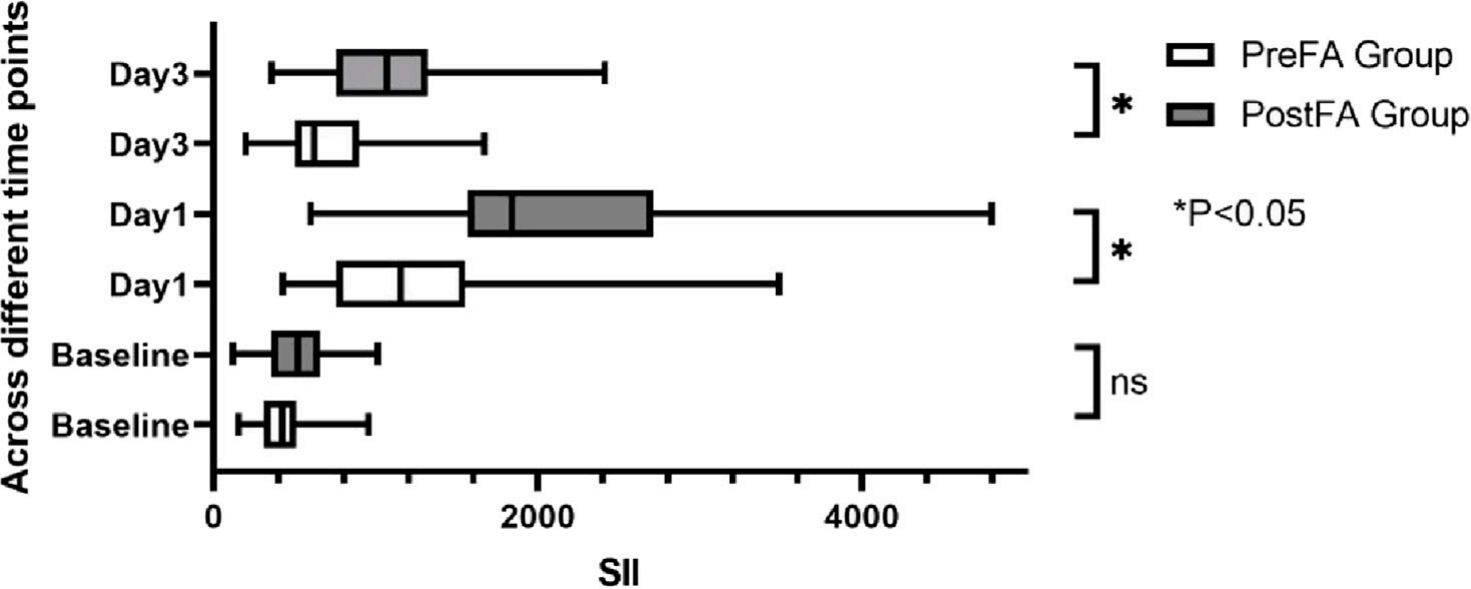
SIl changes across different time points in PreFA and PostFA groups.

**FIGURE 6:**
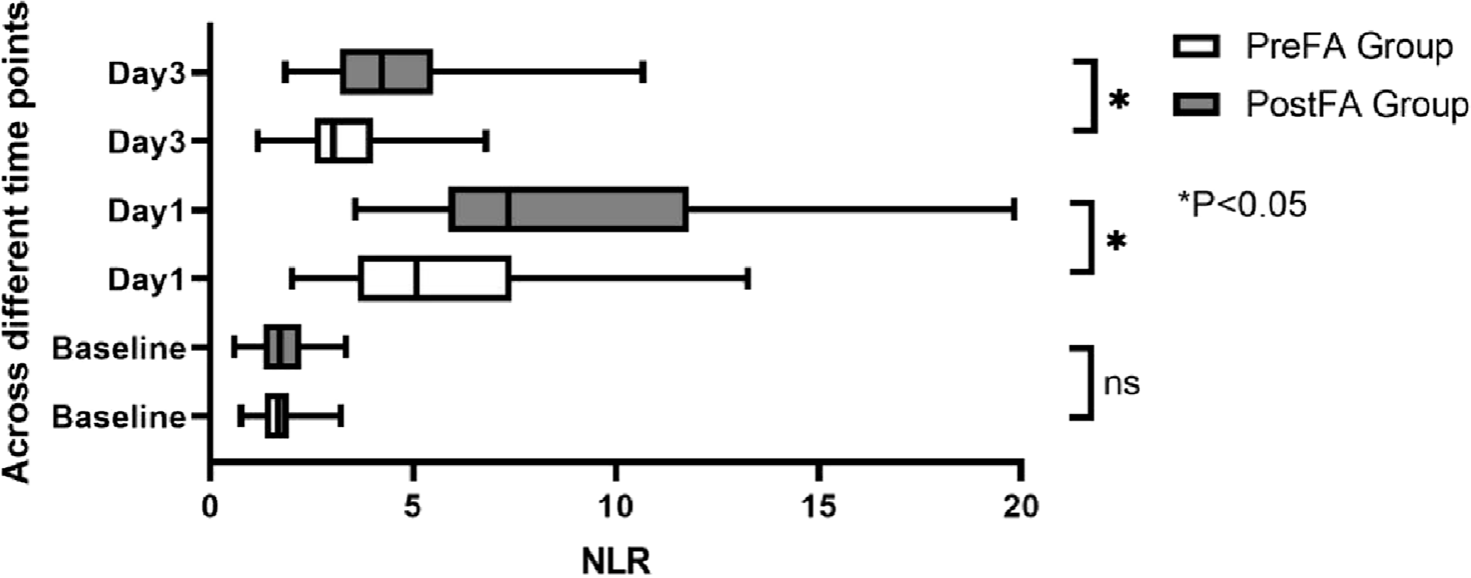
NLR changes across different time points in PreFA and PostFA groups.

**FIGURE 7:**
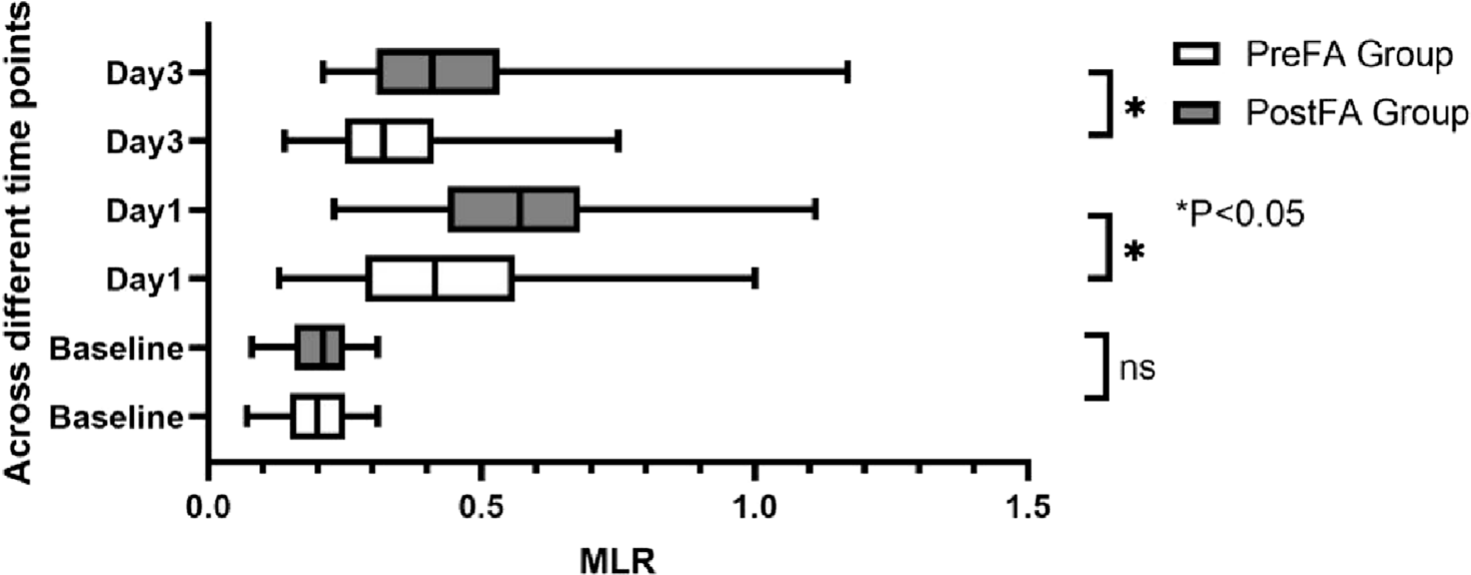
MLR changes across different time points in PreFA and PostFA groups.

There were no significant preoperative differences in SII, NLR, and MLR between the two groups (P = 0.05; 0.53; 0.89). However, all three inflammatory markers significantly increased after surgery (P < 0.001) (Figures 5–7). Additionally, the PreFA group showed significantly lower postoperative SII, NLR, and MLR compared to the PostFA group (P < 0.001) (Figures 5–7).

### 3.5 Comparison of sleep quality and adverse effects between the two groups

**FIGURE 8:**
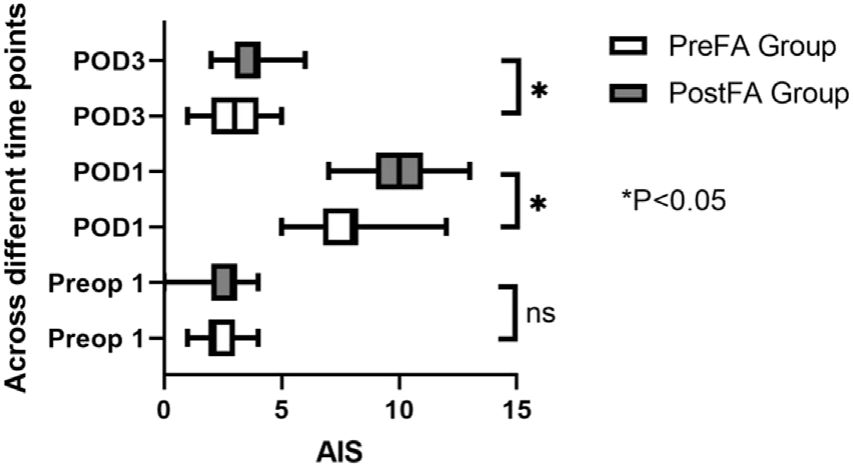
AIS changes at different time points in PreFA and PostFA groups.

There were no significant differences in the AIS score of patients in the PreFA Group and PostFA Group at Sleep-Preop 1 (P = 0.16). The PreFA Group presented a lower AIS score than PostFA Group at Sleep POD 1 and Sleep POD 3 (P < 0.001, P = 0.005 respectively) (Figure 8). The incidence of postoperative nausea and vomiting (PONV) was significantly higher in the PostFA group compared to the PreFA group (50.00% vs. 23.91%, P = 0.010; Table 3).

**TABLE 3:** Comparison of the incidence of postoperative adverse events between the PreFA and PostFA groups.

Postoperative adverse events	PreFA Group (n = 46)	PostFA Group (n = 46)	χ^2^ Value	*P Value*
Nausea and vomiting, n (%)	11 (23.91)	23 (50.00)	6.72	0.01
Dizziness, n (%)	5 (10.87)	7 (15.22)	0.38	0.57
Dyspnea, n (%)	0 (0.00)	3 (6.52)	1.38	0.24
Bradycardia, n (%)	0 (0.00)	3 (6.52)	1.38	0.24
Hypertension, n (%)	2 (4.35)	1 (2.17)	0.00	1.00
Constipation, n (%)	1 (2.17)	1 (2.17)	0.00	1.00
Hypotension, n (%)	2 (4.35)	4 (8.70)	0.18	0.67

This corresponds to an absolute risk reduction (ARR) of 26.09%, with a number needed to treat (NNT) of 3.83—indicating that approximately four patients would need to receive preoperative flurbiprofen axetil to prevent one case of PONV.

Other adverse events, including dizziness, shortness of breath, bradycardia, hypertension, constipation, and hypotension, showed no statistically significant differences between the two groups (P > 0.05; Table 3).

## 4 Discussion

This randomized controlled trial provides evidence that preemptive administration of flurbiprofen axetil can offer clear clinical benefits for patients undergoing laparoscopic gynecological surgery. Compared to postoperative use, preoperative administration of flurbiprofen axetil was associated with enhanced postoperative analgesia (Yamashita et al., 2006; Wang et al., 2017), reduced systemic inflammation (Zhou et al., 2019), and lower opioid consumption (Yamashita et al., 2006). It also showed a trend toward improved sleep quality and fewer complications, although these findings require further validation.

Although prior studies have reported mixed results, our findings support preemptive flurbiprofen axetil as part of multimodal analgesia, emphasizing its role in reducing central sensitization and improving recovers (Halvey et al., 2023). Patients in the preoperative group reported lower pain scores at 24 and 72 h after surgery. They also required less opioid medication and had reduced PCA consumption. These results align with prior research, demonstrating that flurbiprofen axetil mitigates remifentanil-induced hyperalgesia (Zhang et al., 2017). Consistent with earlier studies by Sun et al. (2020), which found positive effects of preoperative NSAID use, our research further expands on this by integrating sleep quality and systemic inflammation as primary outcomes.

Importantly, our findings underscore the critical role of surgical trauma-induced inflammation in the development of postoperative complications. Surgical stress initiates a cascade of neuroendocrine and immune responses, with cytokines such as TNF-α, IL-6, and CRP playing central roles (Ivascu et al., 2024).

Recent studies have suggested that elevated systemic inflammation is linked to disruptions in sleep architecture (Engert and Besedovsky, 2025) with inflammatory markers also contributing to hyperalgesia and poor postoperative recovery (Savic Vujovic et al., 2023; Cocea and Stocia, 2024). Our results corroborate this connection: patients in the preoperative group demonstrated significantly lower levels of SII, NLR, and MLR—biomarkers increasingly recognized as sensitive indicators of perioperative immune dysregulation.

The improvement in postoperative sleep quality may be explained by the combined effects of better analgesia and reduced inflammation. Sleep disruption was prevalent in both groups, particularly on postoperative days 1 and 3 as shown by elevated AIS scores; however, patients in the preoperative group consistently reported better sleep. This reinforces the notion that pain and inflammation are key modifiable factors contributing to postoperative sleep disturbances. Moreover, opioids are known to suppress REM and slow-wave sleep (Wang and Teichtahl, 2007), and reduced opioid consumption in the preoperative group was likely contributed to enhanced sleep quality. The outcomes are in agreement with those documented by Knill et al., who demonstrated that postoperative REM sleep was inversely proportional to morphine use, with REM duration gradually increasing as opioid use declined (Knill et al., 1990). Flurbiprofen axetil’s pharmacological properties make it particularly effective for preemptive analgesia (Wang et al., 2017). As a lipid microsphere formulation, it allows for targeted and sustained drug delivery, prolonging analgesic effects while minimizing respiratory depression (Gu et al., 2020). Our study confirms that flurbiprofen axetil effectively reduces perioperative opioid requirements and alleviates inflammatory overactivation, promoting a smoother and faster recovery.

Beyond pain and inflammation, it is also important to recognize the broader impact of postoperative sleep disturbances and complications such as nausea and vomiting on recovery quality. These adverse outcomes can impair mobilization, delay wound healing, prolong hospital stay, and reduce overall patient satisfaction. The lower incidence of such complications observed in the preoperative group suggests that early administration of flurbiprofen axetil may help improve not only immediate perioperative outcomes but also the overall quality of recovery.

Despite these strengths, several limitations must be acknowledged. Sleep quality was assessed using the subjective AIS scale without objective validation through polysomnography or actigraphy. Although environmental variables such as noise, light, and nursing interventions were minimized, they could not be entirely controlled.

Furthermore, the study took place at a single center with a uniform group of female patients undergoing laparoscopic uterine procedures, which restricts the broader applicability of the findings. From a clinical perspective, these findings support incorporating flurbiprofen axetil into preemptive multimodal analgesic protocols. This strategy improves pain control, reduces systemic inflammation, and enhances postoperative sleep—thereby potentially accelerating recovery and reducing opioid dependence (Wang et al., 2012; Zhou et al., 2019). Future studies should involve multicenter trials encompassing various surgical groups, with a focus on objective monitoring of sleep, pain, and long-term outcomes to evaluate chronic pain and functional recovery.

To summarize, our results demonstrate that preemptive flurbiprofen axetil serves as a multifunctional adjunct in perioperative care, offering simultaneous benefits in pain control, attenuation of systemic inflammation—as evidenced by reductions in SII, NLR, and MLR—and improved sleep maintenance, thereby supporting enhanced postoperative outcomes in laparoscopic gynecological procedures and potentially other minimally invasive surgeries.

## Data Availability

The original contributions presented in the study are included in the article, further inquiries can be directed to the corresponding author.
